# Supplementary material for: Natural Occurrence of Conventional and Emerging Fusarium Mycotoxins in Freshly Harvested Wheat Samples in Xinjiang, China
Source: Toxins (Basel). 2025 Dec 10;17(12):591. doi: 10.3390/toxins17120591 (PMC12737364; doi:10.3390/toxins17120591)
Supplement: Supplementary file 1 [file toxins-17-00591-s001.zip › toxins-3993991-supplementary/toxins-3993991-supplementary.pdf]

Supplementary Materials: Natural Occurrence of Conventional and Emerging *Fusarium* Mycotoxins in the Freshly Harvested Wheat Samples in Xinjiang, China

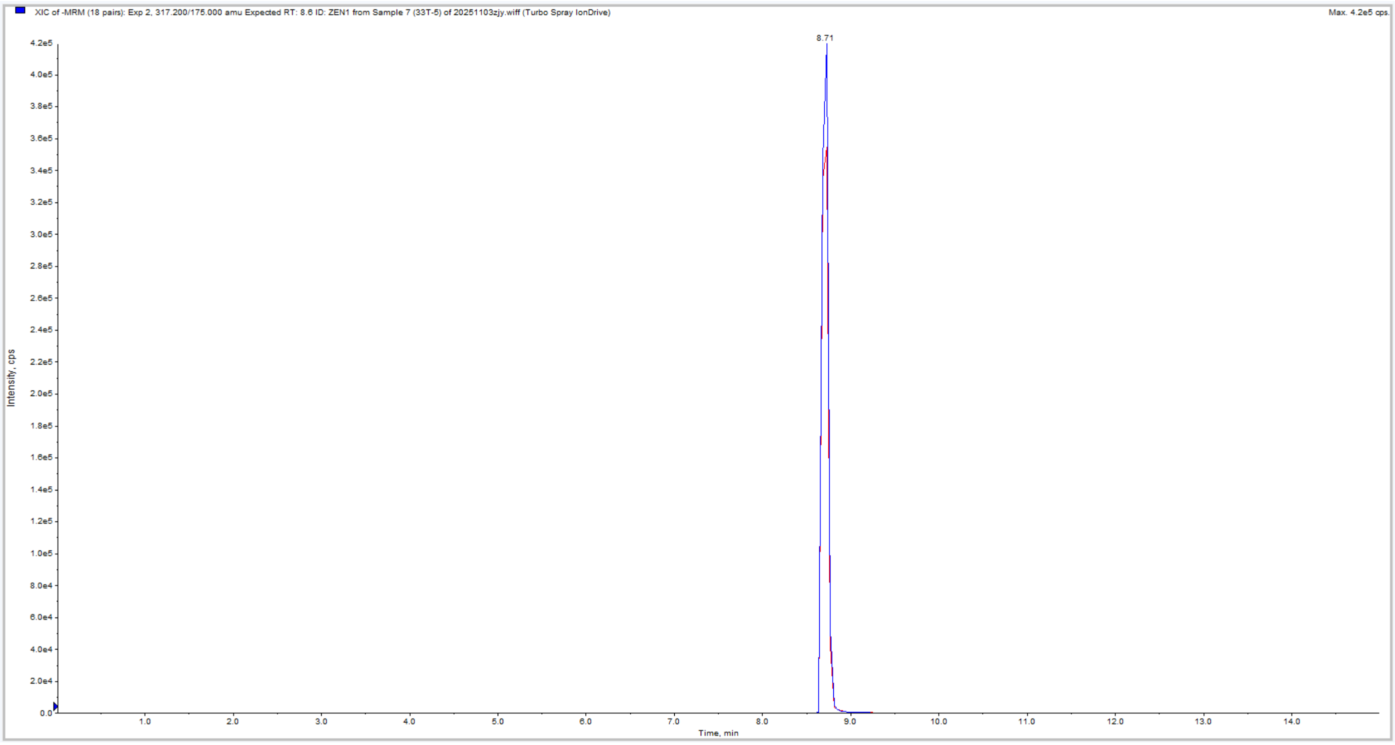

Figure S1. Extracted ion chromatogram (XIC) of ZEN obtained in negative MRM mode (-MRM), showing the signal at *m/z* 317.200/175.000 with an expected retention time of 8.71 min. Data correspond to ZEN (40 μg/kg) in the mixed standard solution.

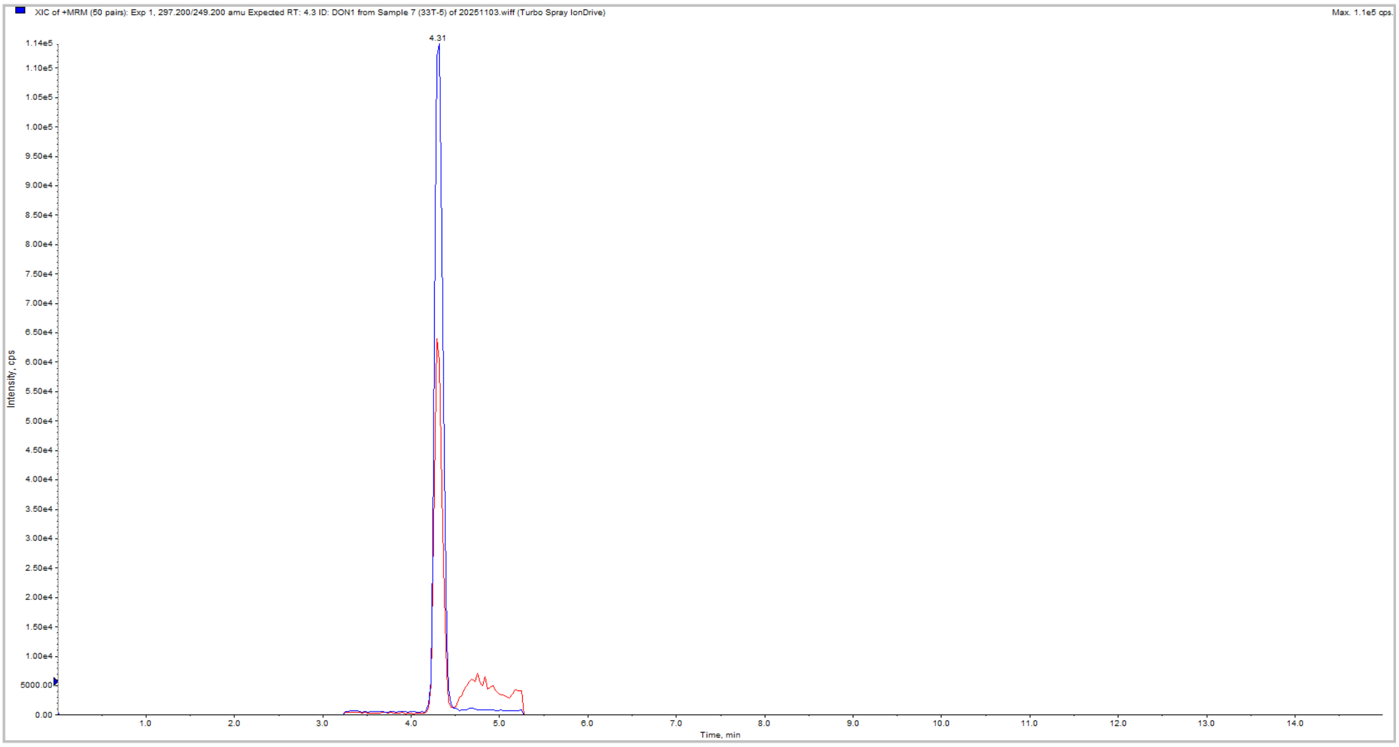

Figure S2. XIC of DON obtained in positive MRM mode (+MRM), showing the signal at *m/z* 297.200/249.200 with an expected retention time of 4.31 min. Data correspond to DON (400 μg/kg) in the mixed standard solution.

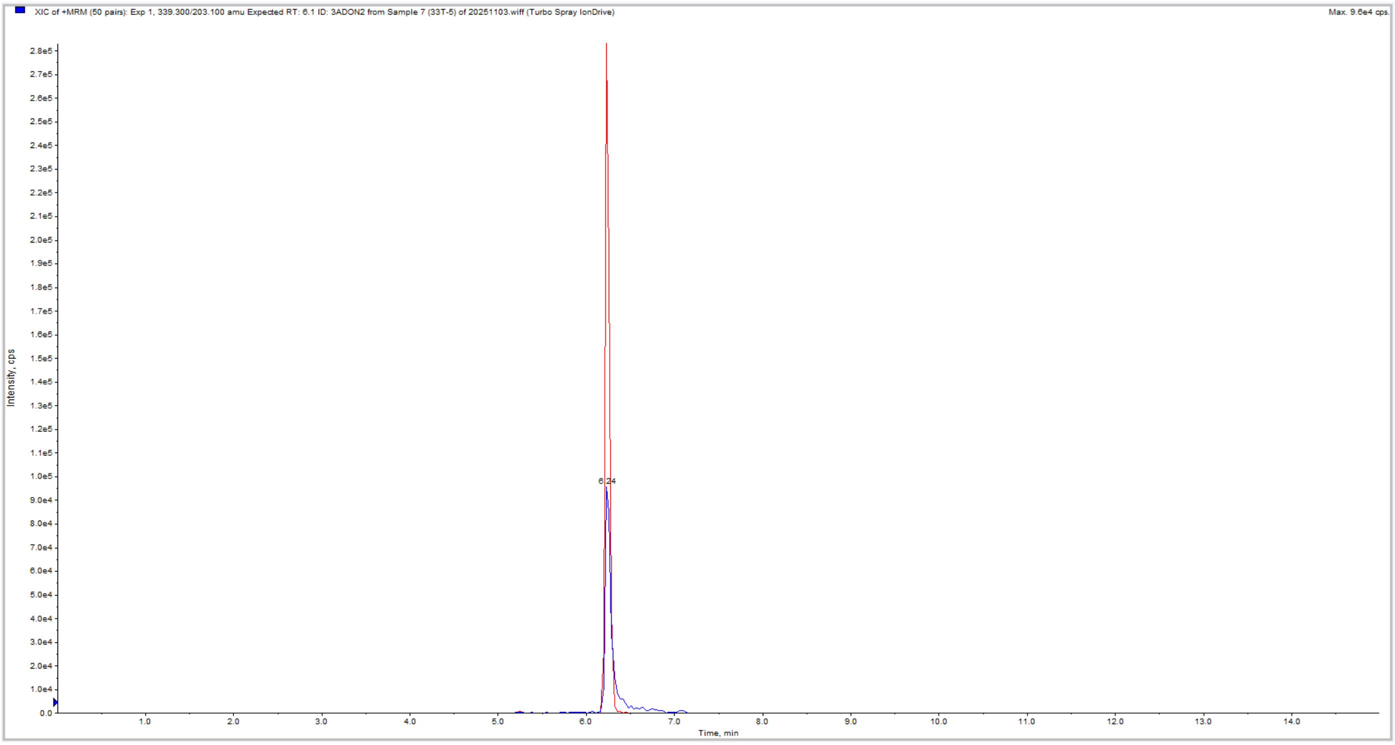

Figure S3. XIC of 3ADON obtained in +MRM, showing the signal at *m/z* 339.300/203.100 with an expected retention time of 6.24 min. Data correspond to 3ADON (400 μg/kg) in the mixed standard solution.

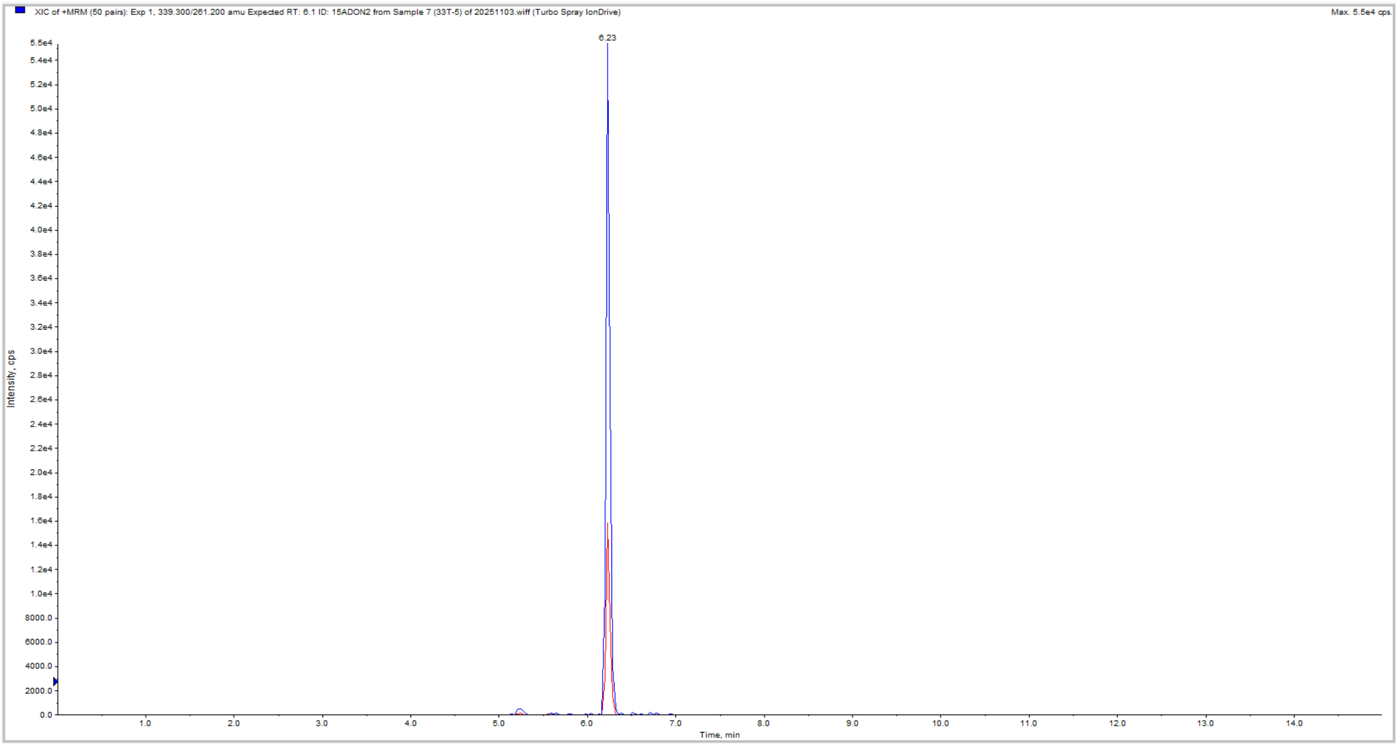

Figure S4. XIC of 15ADON obtained in +MRM, showing the signal at *m/z* 339.300/261.200 with an expected retention time of 6.23 min. Data correspond to 15ADON (400 μg/kg) in the mixed standard solution.

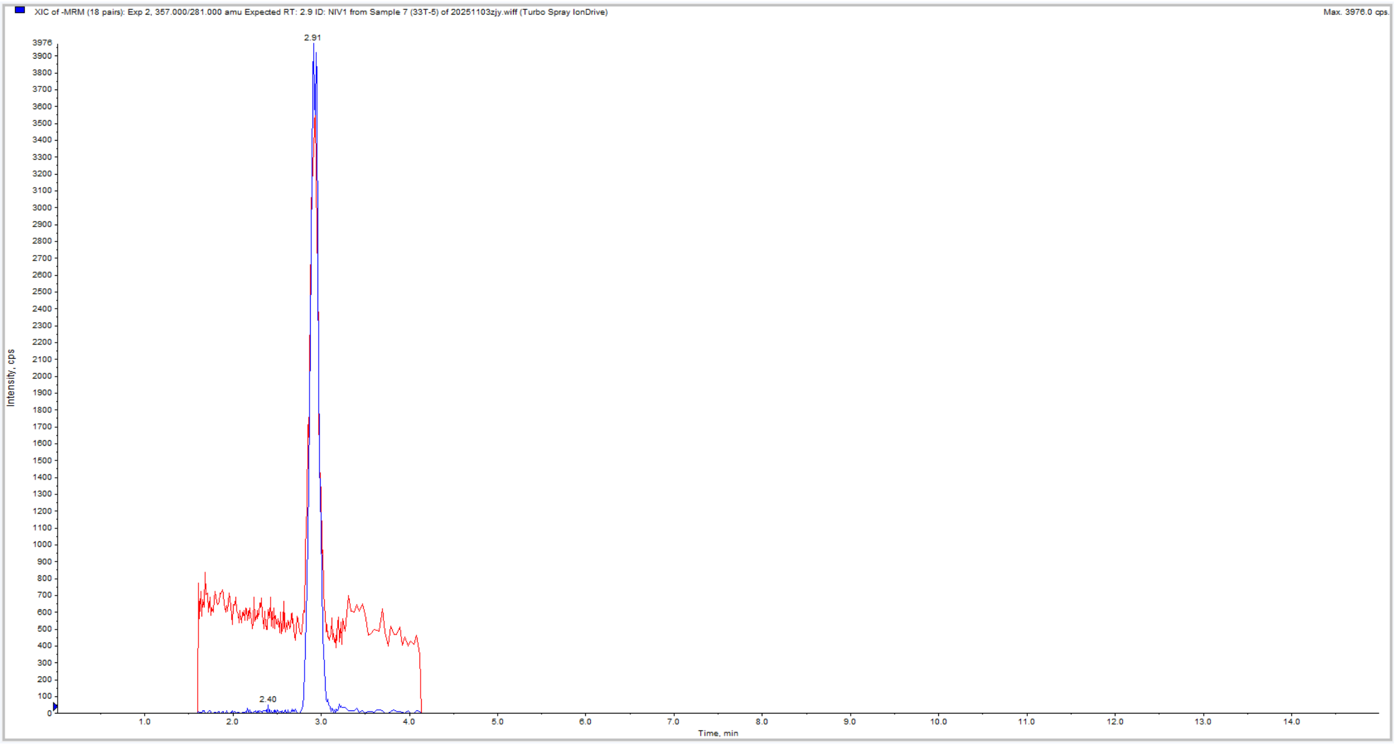

Figure S5. XIC of NIV obtained in -MRM, showing the signal at *m/z* 357.000/281.000 with an expected retention time of 2.91 min. Data correspond to NIV (200 μg/kg) in the mixed standard solution.

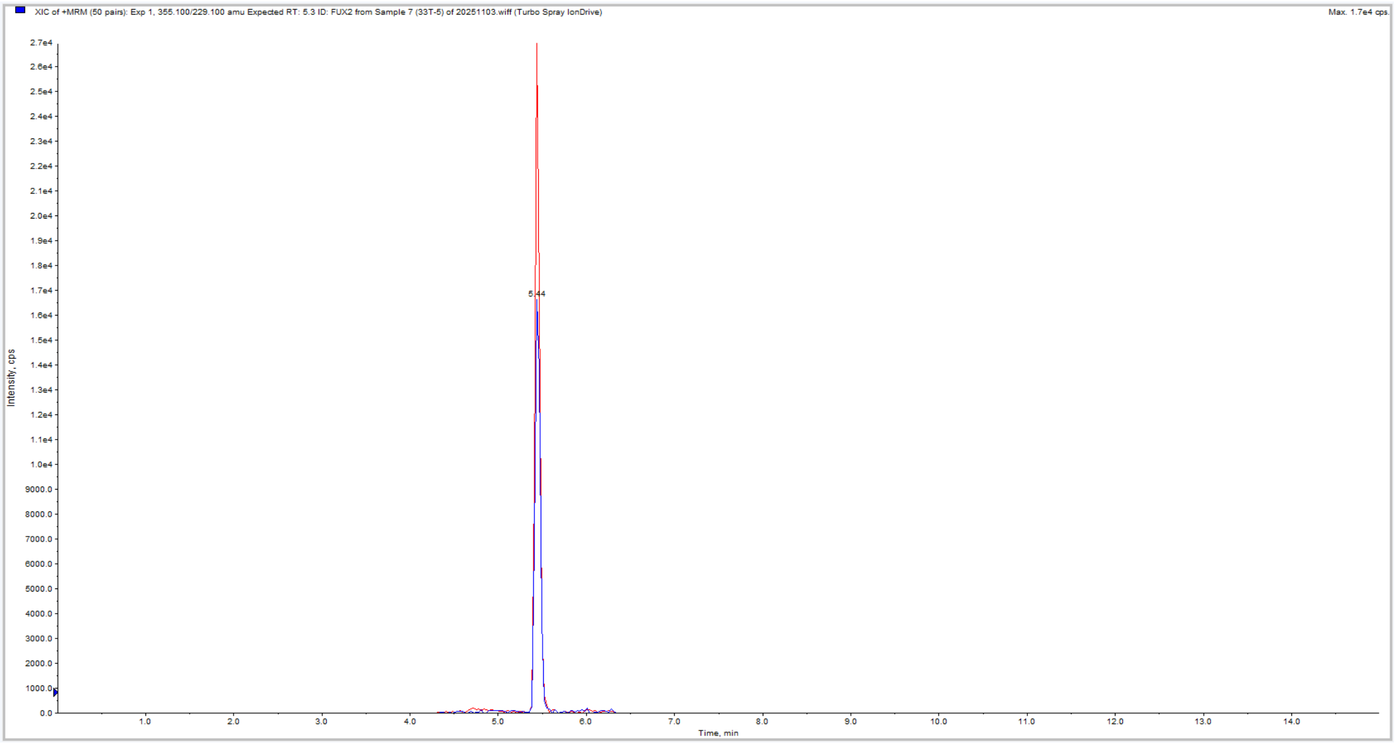

Figure S6. XIC of 4ANIV obtained in +MRM, showing the signal at *m/z* 335.100/229.100 with an expected retention time of 5.44 min. Data correspond to 4ANIV (400 µg/kg) in the mixed standard solution.

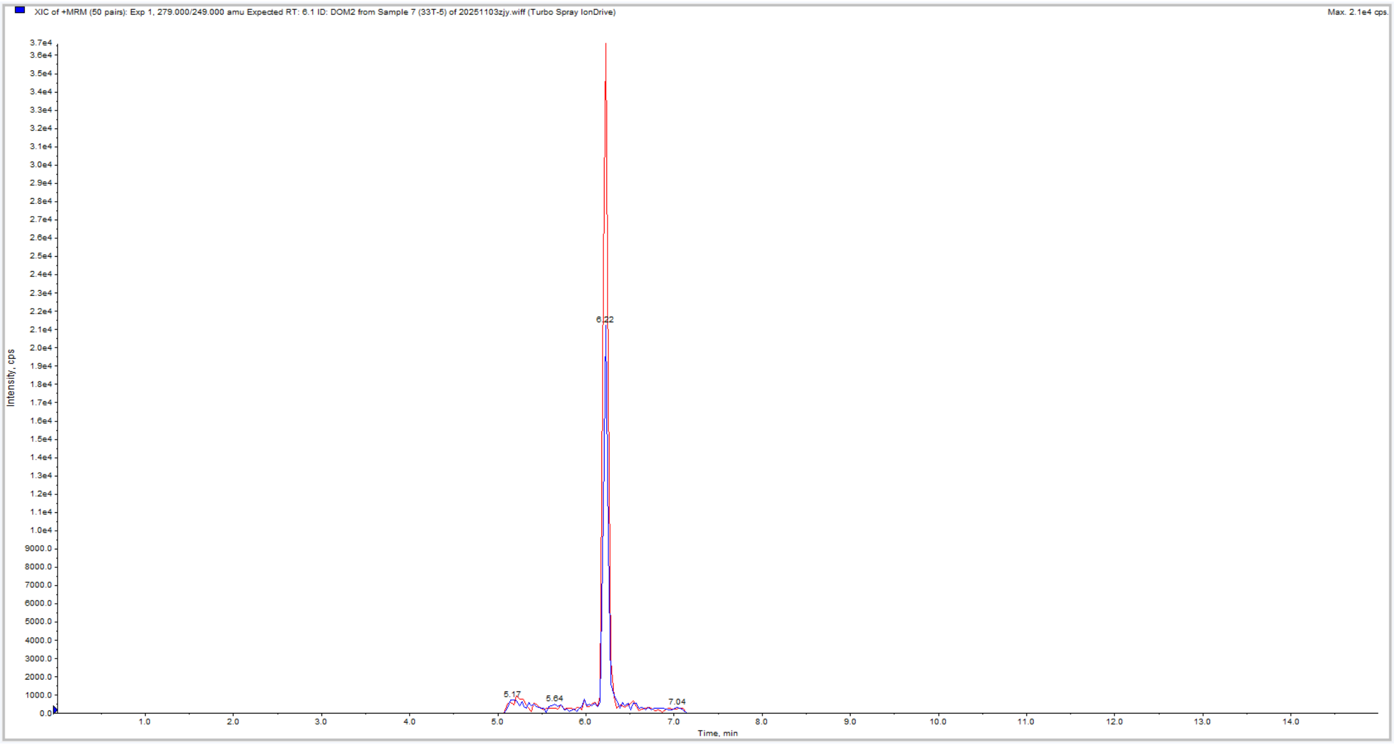

Figure S7. XIC of DOM obtained in +MRM, showing the signal at *m/z* 279.000/249.000 with an expected retention time of 6.22 min. Data correspond to DOM (200 µg/kg) in the mixed standard solution.

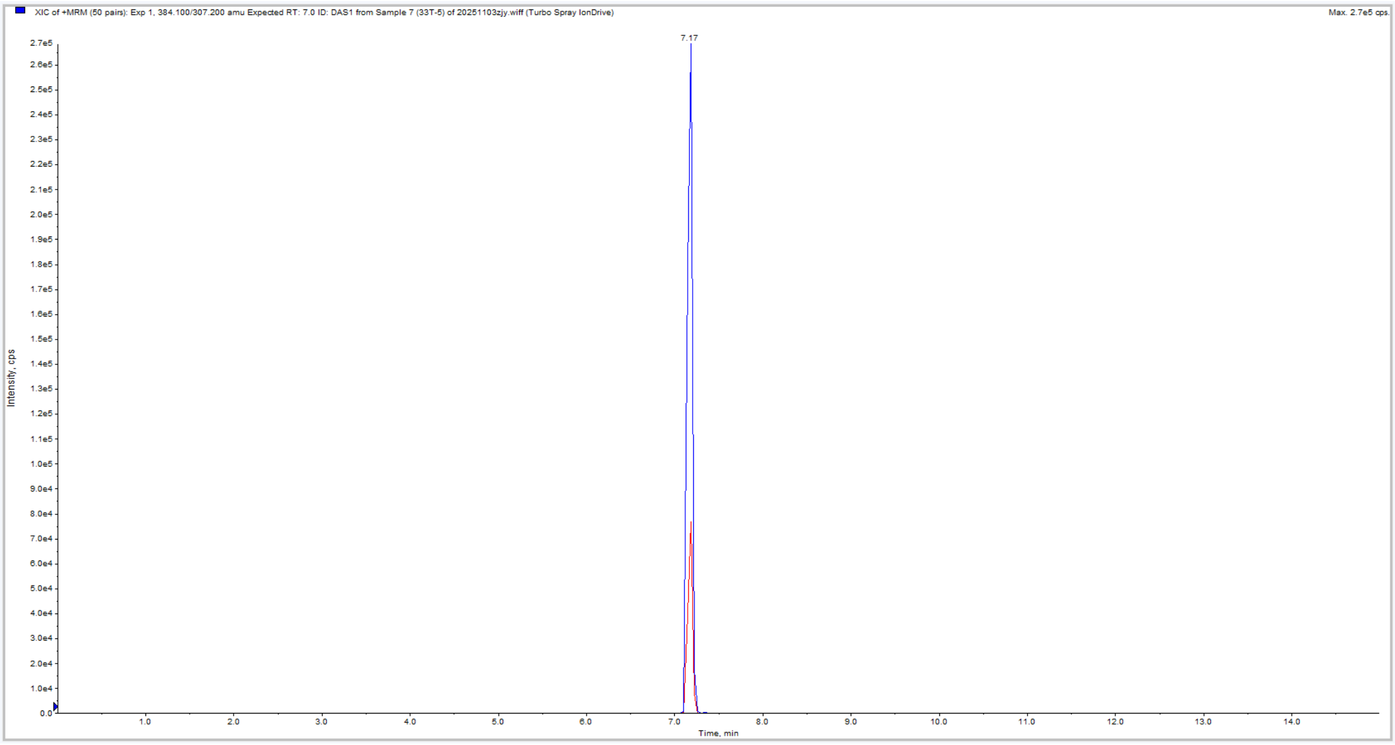

Figure S8. XIC of DAS obtained in +MRM, showing the signal at *m*/*z* 384.100/307.200 with an expected retention time of 7.17 min. Data correspond to DAS (200 μg/kg) in the mixed standard solution.

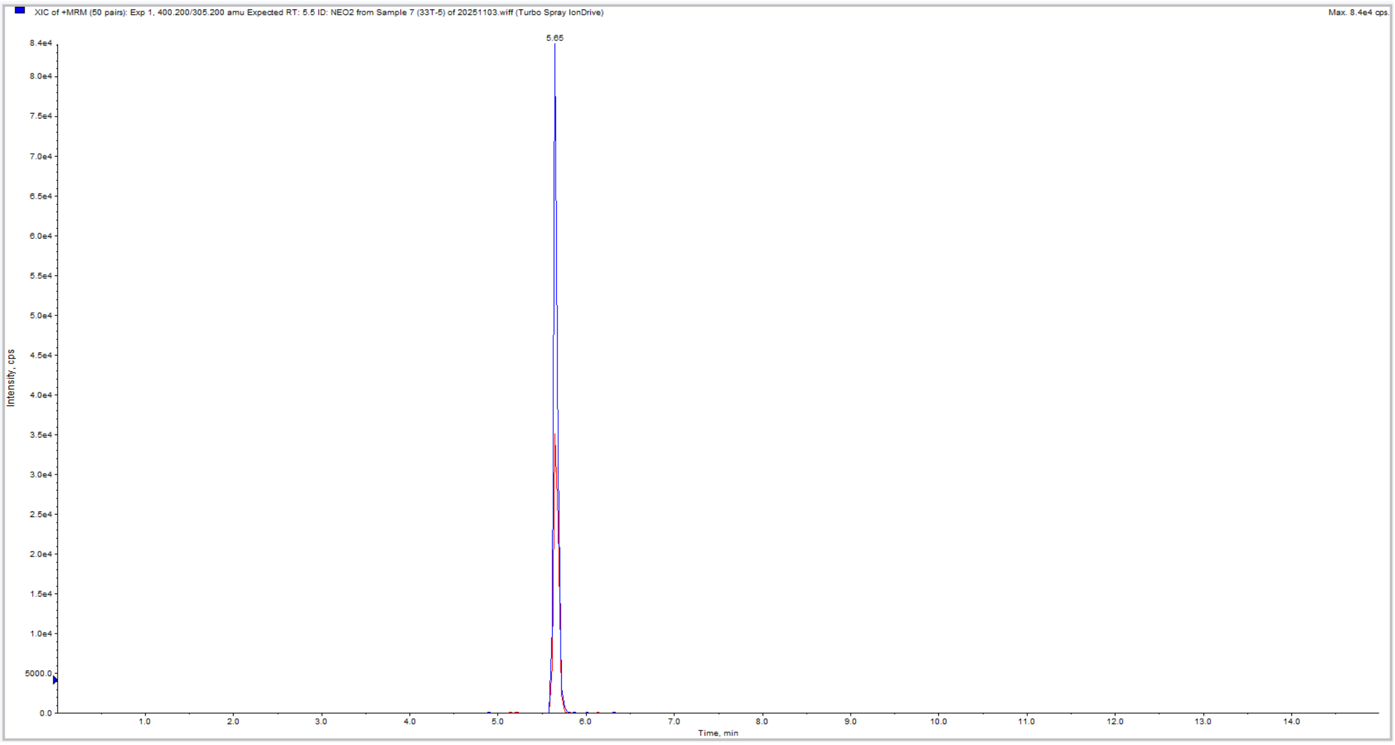

Figure S9. XIC of NEO obtained in +MRM, showing the signal at *m*/*z* 400.200/305.200 with an expected retention time of 5.65 min. Data correspond to NEO (200 μg/kg) in the mixed standard solution.

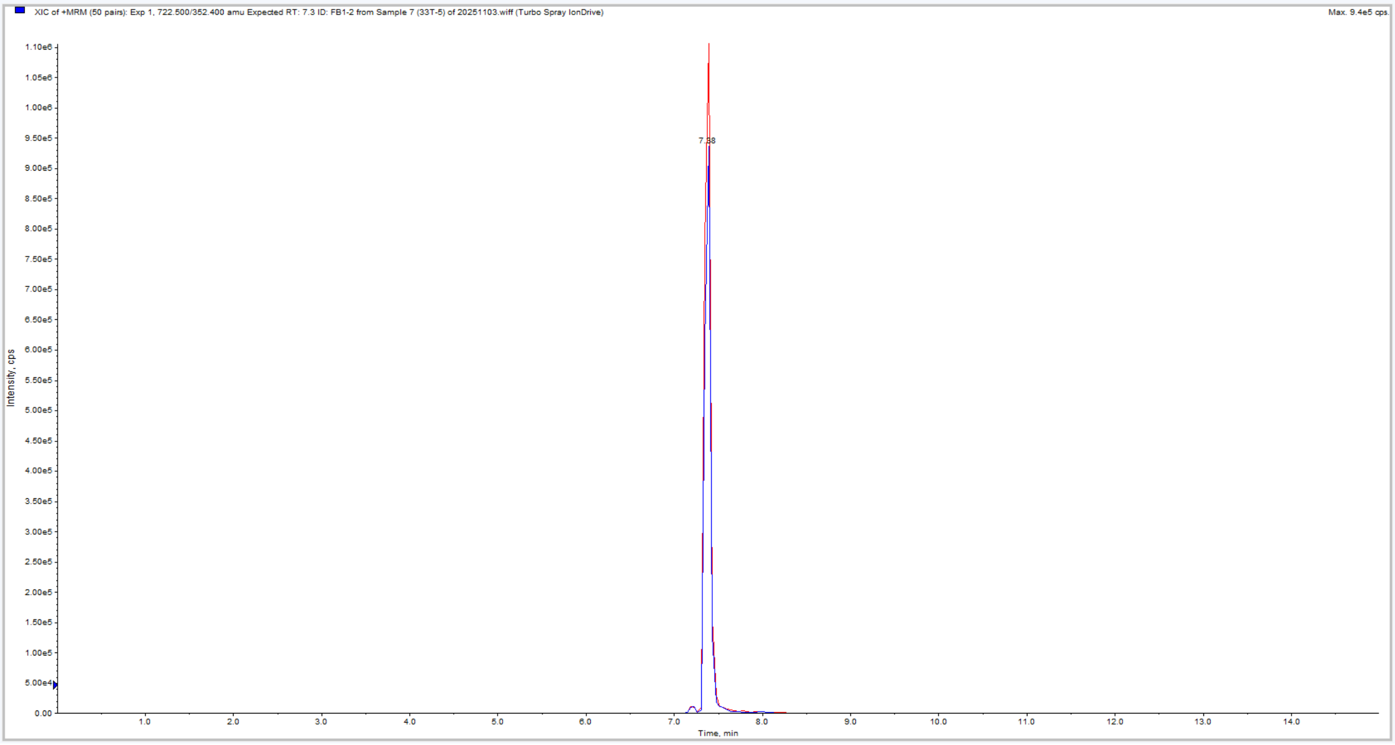

Figure S10. XIC of FB_1 obtained in +MRM, showing the signal at *m*/*z* 722.500/352.400 with an expected retention time of 7.38 min. Data correspond to FB_1 (200 µg/kg) in the mixed standard solution.

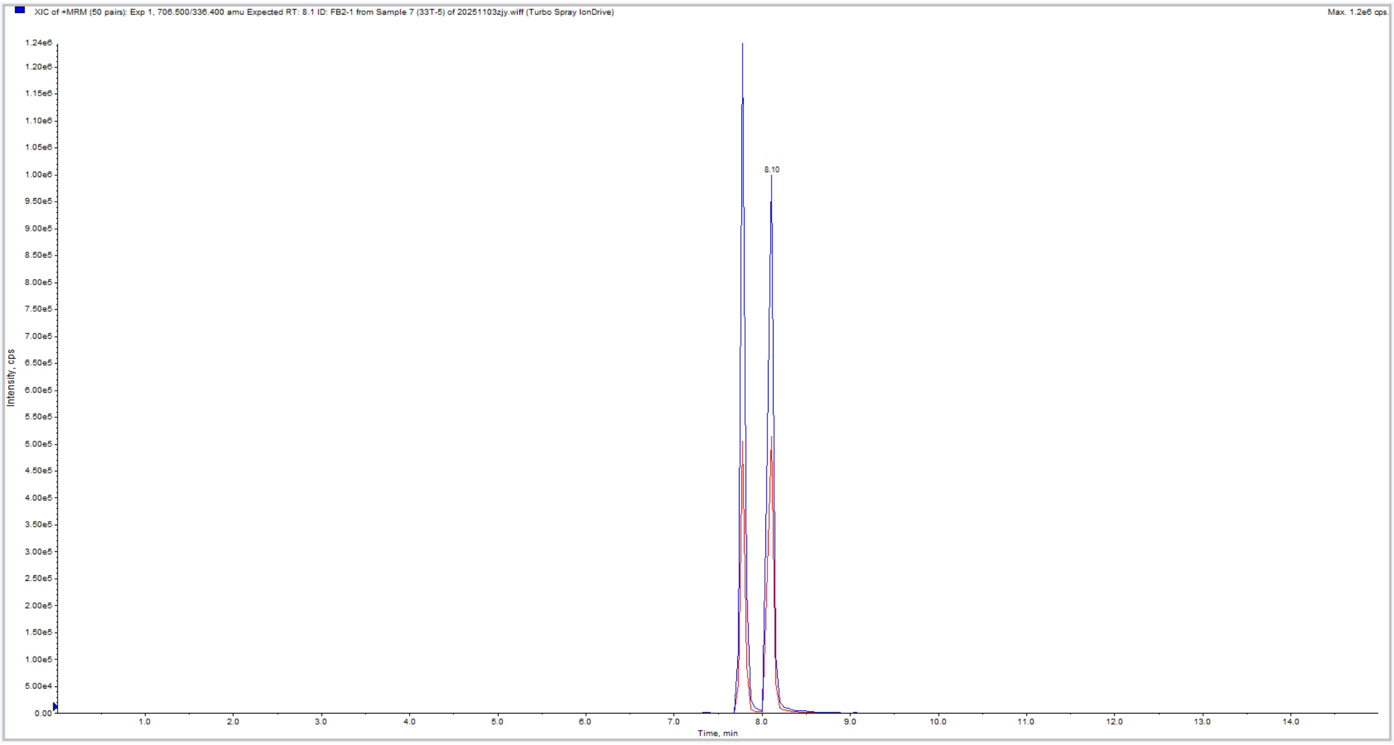

Figure S11. XIC of FB_2 obtained in +MRM, showing the signal at *m*/*z* 706.500/336.400 with an expected retention time of 8.10 min. Data correspond to FB_2 (200 µg/kg) in the mixed standard solution.

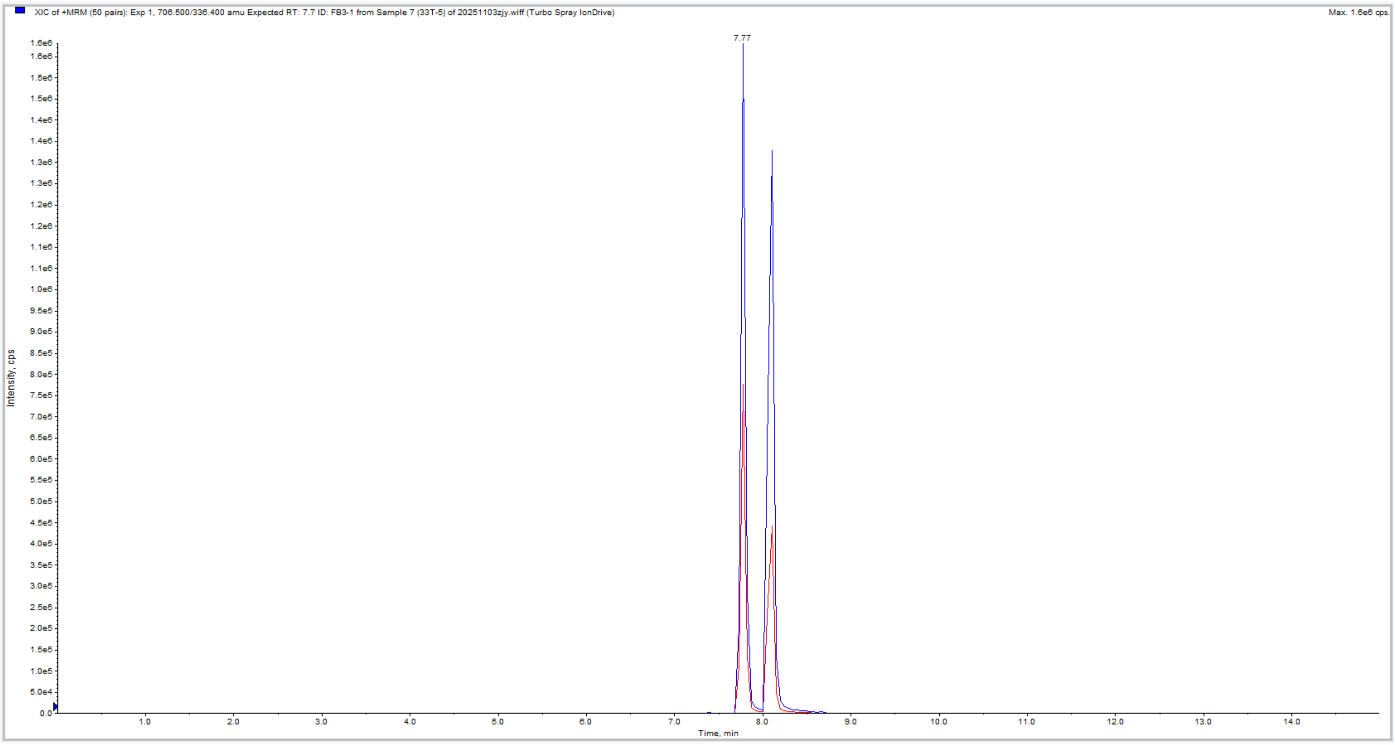

Figure S12. XIC of FB3 obtained in +MRM, showing the signal at *m/z* 706.500/336.400 with an expected retention time of 7.77 min. Data correspond to FB3 (200 µg/kg) in the mixed standard solution.

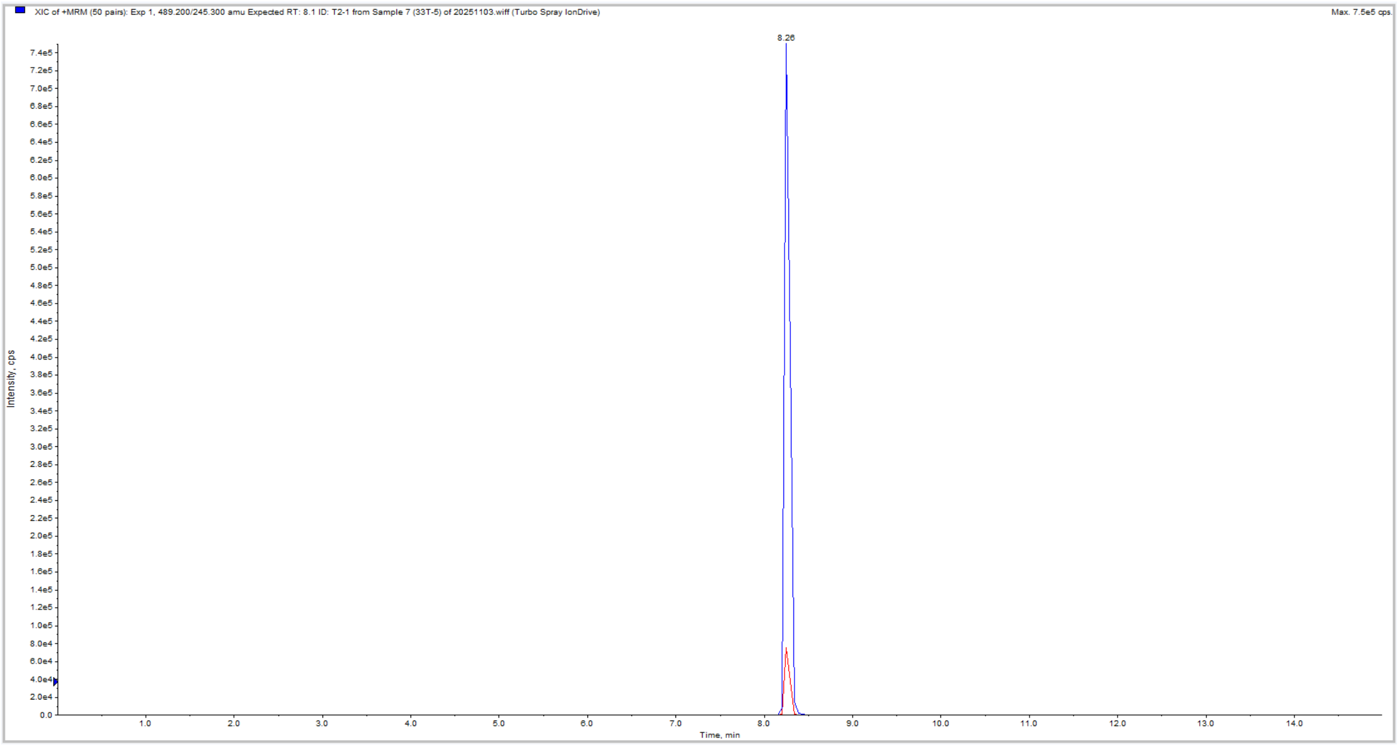

Figure S13. XIC of T-2 obtained in +MRM, showing the signal at *m/z* 489.200/245.300 with an expected retention time of 8.26 min. Data correspond to T-2 (200 µg/kg) in the mixed standard solution.

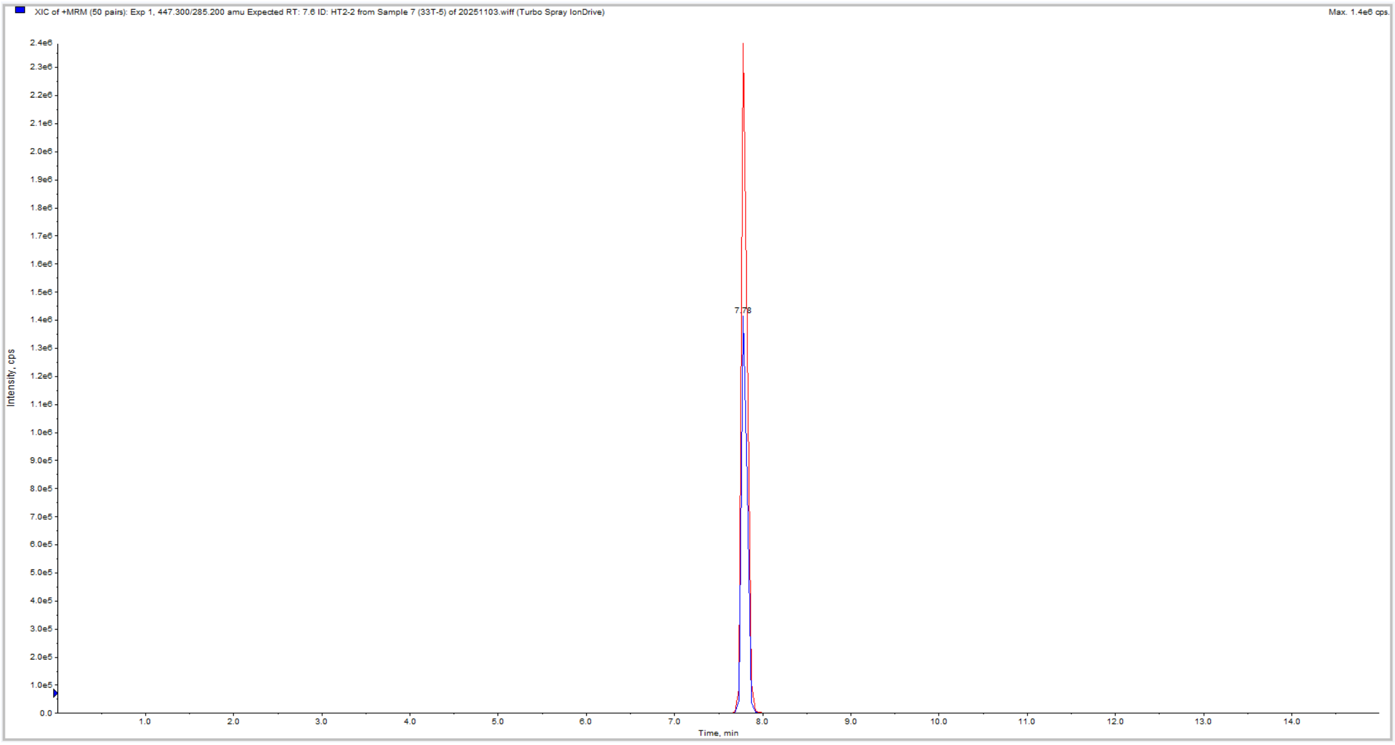

Figure S14. XIC of HT-2 obtained in +MRM, showing the signal at *m/z* 447.300/285.200 with an expected retention time of 8.26 min. Data correspond to HT-2 (200 µg/kg) in the mixed standard solution.

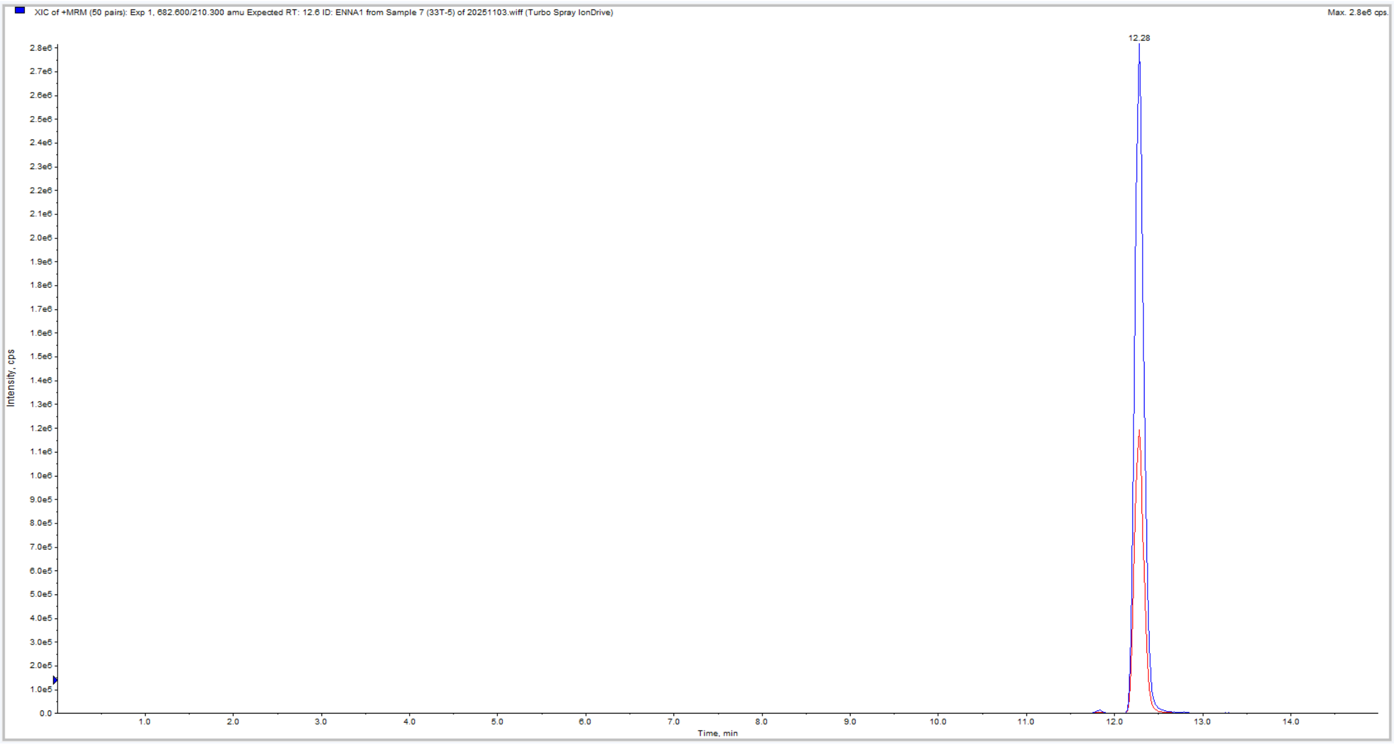

Figure S15. XIC of ENNA obtained in +MRM, showing the signal at *m/z* 682.600/210.300 with an expected retention time of 12.28 min. Data correspond to ENNA (200 µg/kg) in the mixed standard solution.

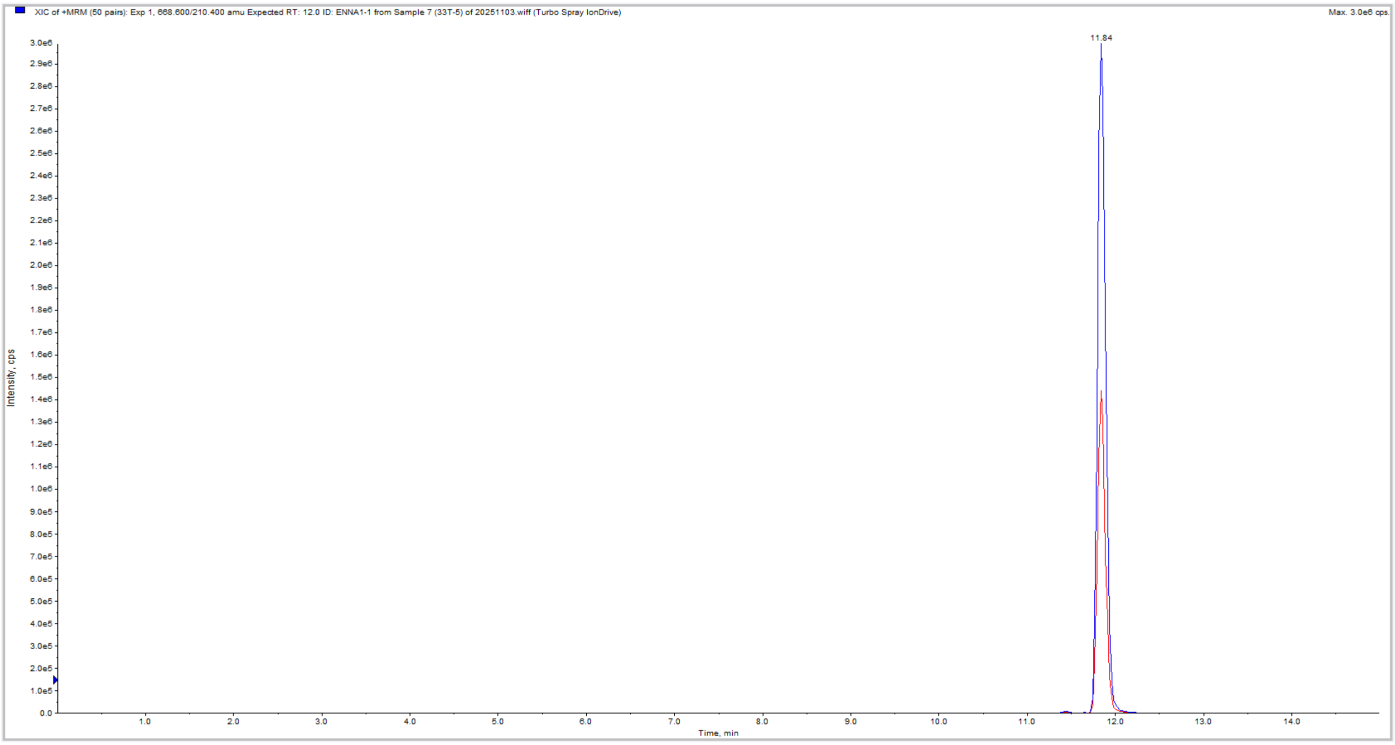

Figure S16. XIC of ENNA1 obtained in +MRM, showing the signal at *m*/*z* 668.600/210.400 with an expected retention time of 11.84 min. Data correspond to ENNA1 (200 µg/kg) in the mixed standard solution.

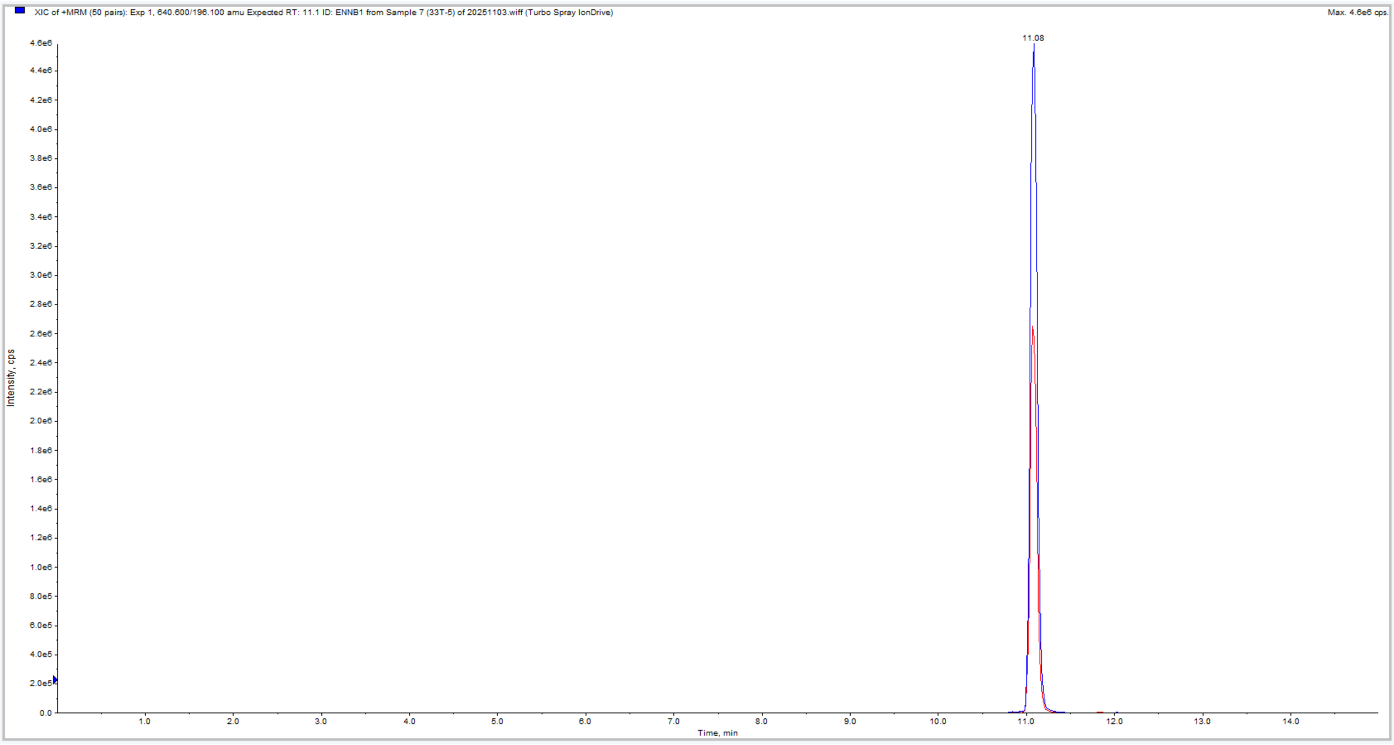

Figure S17. XIC of ENNB obtained in +MIC, showing the signal at *m*/*z* 640.600/296.100 with an expected retention time of 11.08 min. Data correspond to ENNB (200 µg/kg) in the mixed standard solution.

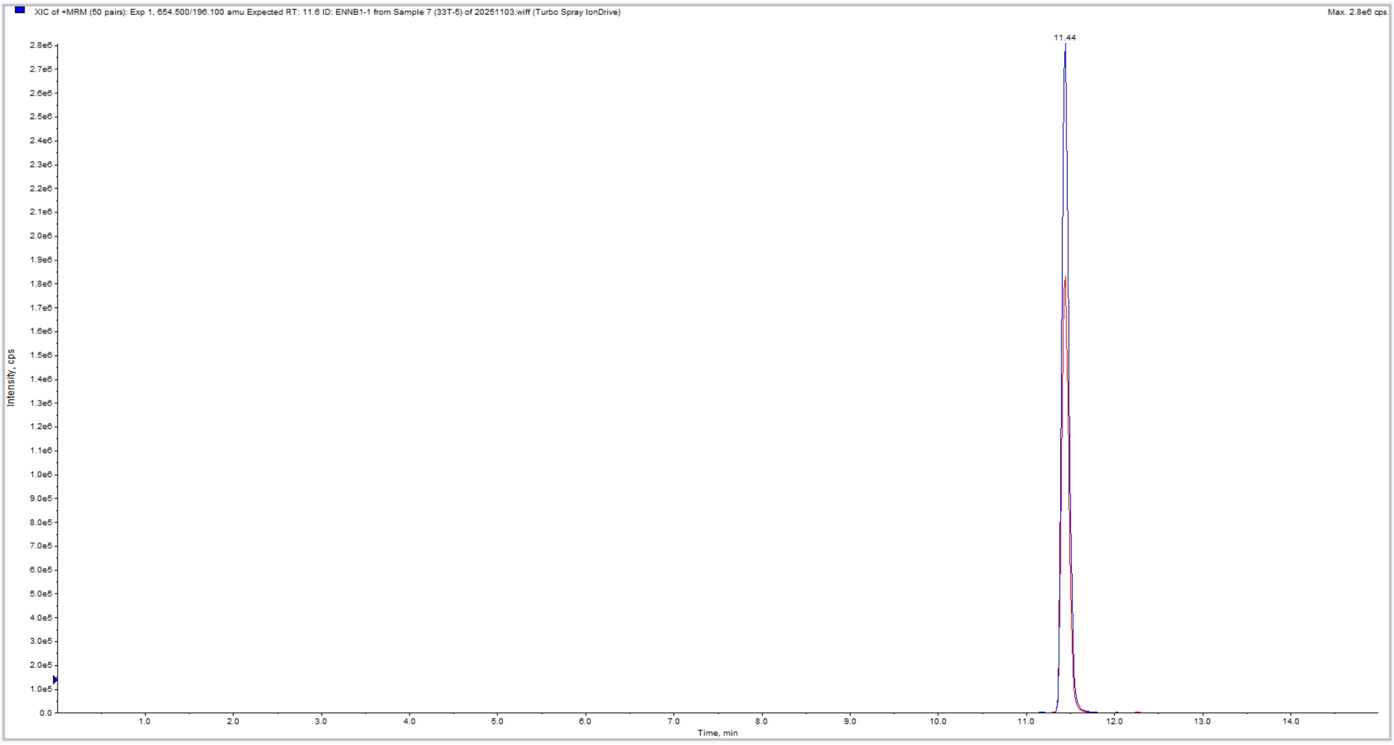

Figure S18. XIC of ENNB1 obtained in +MIC, showing the signal at *m/z* 654.500/196.100 with an expected retention time of 11.44 min. Data correspond to ENNB1 (200 µg/kg) in the mixed standard solution.

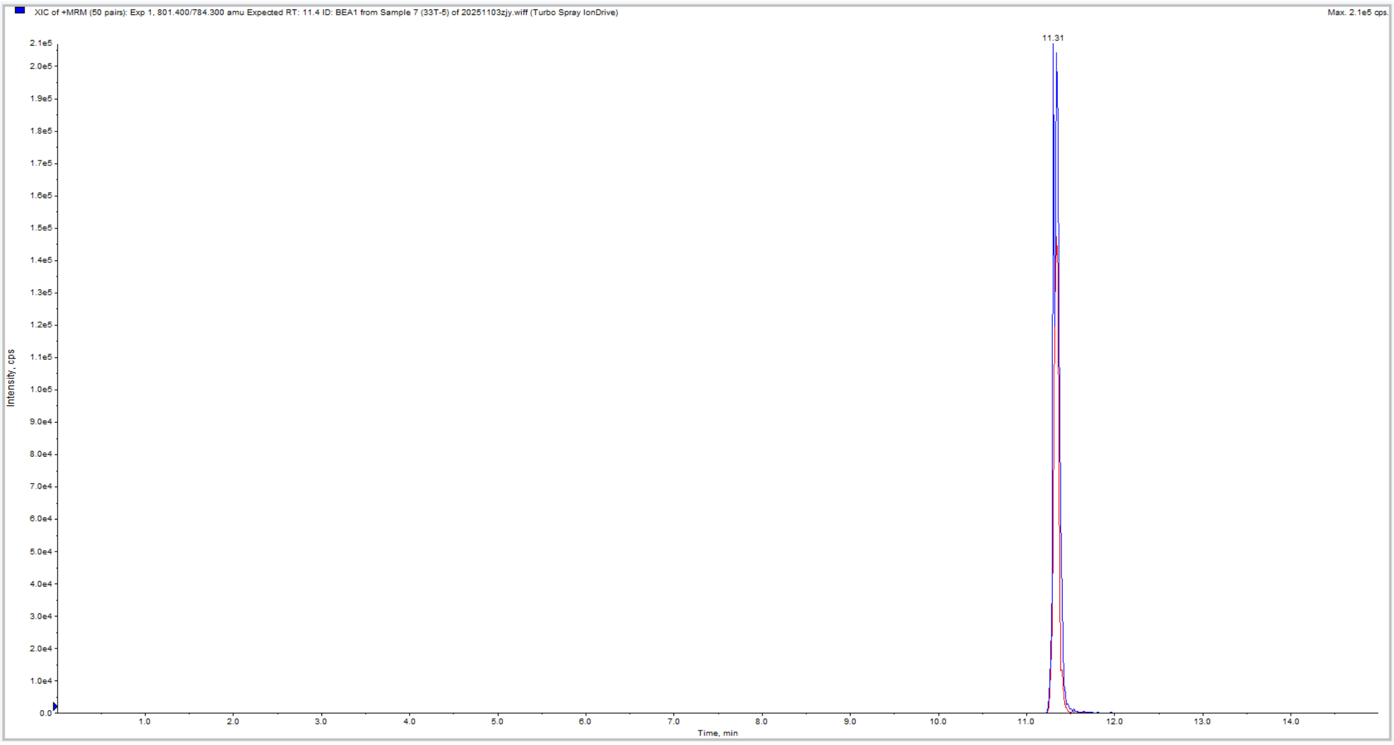

Figure S19. XIC of BEA obtained in +MIC, showing the signal at *m/z* 801.400/784.300 with an expected retention time of 11.08 min. Data correspond to BEA (200 µg/kg) in the mixed standard solution.
